# A Finger Vein Feature Extraction Method Incorporating Principal Component Analysis and Locality Preserving Projections

**DOI:** 10.3390/s22103691

**Published:** 2022-05-12

**Authors:** Dingzhong Feng, Shanyu He, Zihao Zhou, Ye Zhang

**Affiliations:** 1 College of Mechanical Engineering, Zhejiang University of Technology, Hangzhou 310014, China; fdz@zjut.edu.cn (D.F.); 2111902007@zjut.edu.cn (S.H.); 2111902078@zjut.edu.cn (Z.Z.); 2 Zhejiang Jinghong Intelligent Technology Co., Ltd., Jinyun 321400, China

**Keywords:** finger vein recognition, biometric recognition, feature extraction method, algorithm

## Abstract

In the field of biometric recognition, finger vein recognition has received widespread attention by virtue of its advantages, such as biopsy, which is not easy to be stolen. However, due to the limitation of acquisition conditions such as noise and illumination, as well as the limitation of computational resources, the discriminative features are not comprehensive enough when performing finger vein image feature extraction. It will lead to such a result that the accuracy of image recognition cannot meet the needs of large numbers of users and high security. Therefore, this paper proposes a novel feature extraction method called principal component local preservation projections (PCLPP). It organically combines principal component analysis (PCA) and locality preserving projections (LPP) and constructs a projection matrix that preserves both the global and local features of the image, which will meet the urgent needs of large numbers of users and high security. In this paper, we apply the Shandong University homologous multi-modal traits (SDUMLA-HMT) finger vein database to evaluate PCLPP and add “Salt and pepper” noise to the dataset to verify the robustness of PCLPP. The experimental results show that the image recognition rate after applying PCLPP is much better than the other two methods, PCA and LPP, for feature extraction.

## 1. Introduction

### 1.1. Motivation

With the development of the digital economy and network security systems, the authentication of identity information is ubiquitous. Identity verification is required in daily life scenarios such as unlocking cell phones, logging into personal electronic accounts, using ATMs, and e-commerce. Traditional authentication technologies such as passwords and PINs [1] have some disadvantages, for instance, low security and being easy to forget and lose, making it difficult to meet the security needs in many application scenarios. Biometric identification technology [2] has received more and more attention in recent years because of its advantages, such as living identification, being difficult to steal, and having unique characteristics.

Biometric identification is a technology that allows personal identification by computer processing inherent physiological characteristics or behavioral traits of the human body. These characteristics are usually obtained by biological sensors, for example, optical sensors, acoustic sensors, etc. Table 1 compares the features of some mainstream biometric technologies in terms of accuracy, stability, recognition speed, etc.

Finger vein recognition is a bioassay technology, and the finger vein characteristics of different finger individuals are unique, which enables the high-accuracy identification of a large number of users [3]. In addition, as a non-contact biometric identification method, finger vein recognition is clean and hygienic. It means that the breakage and dirt of the epidermis will not affect the accuracy of recognition [4]. It is due to these advantages that research and development regarding finger vein recognition technology has received wide attention.

Although finger vein recognition has advantages over other biometric technologies, the fact is that it is not yet mature. Finger vein recognition techniques based on traditional methods, such as SIFT [5], Gabor [6], and maximum curvature [7], are not suitable for a large number of users and have been gradually phased out. Corresponding to these traditional methods are the image processing methods based on machine learning [8,9,10]. Among them, the deep learning image processing method [11] can obtain higher accuracy, but it often requires a large number of training samples, so it is not widely used at present. However, the widely used finger vein datasets are all small sample datasets. Therefore, it is urgent to find a method that can obtain accurate finger vein features even in small samples of finger vein datasets.

### 1.2. Contribution

The contributions of this work are threefold:

(1) We proposed a finger vein feature extraction algorithm, which combines principal component analysis and local preservation projection. It can effectively obtain global key features and local key features of finger vein images for feature classification.
(2) The algorithm we proposed has good recognition accuracy in ROI images that have not been pre-processed with image enhancement.
(3) The algorithm is robust for “Salt and pepper” noise and Gaussian noise.

The rest of this paper is organized as follows. Previous studies are described in Section 2. In Section 3, the proposed method is described. Comparative experiments and experimental results with analysis are described in Section 4. Finally, the conclusions of this paper are provided in Section 5.

## 2. Literature Review

Traditional finger vein recognition methods require image preprocessing and feature extraction first. To reduce the computational cost, many researchers perform dimensionality reduction on the images by PCA before feature extraction. Wu and Liu [12] applied PCA for a feature dimension reduction on finger vein images. After comparing the adaptive neuro-fuzzy inference system (ANFIS) classification with the back-propagation (BP) neural network classification, they found that the former was better than the latter. Later, they [13] performed a feature extraction on images by combining PCA and linear discriminant analysis (LDA). After applying the ANFIS and support vector machine (SVM) for classification, respectively, they finally concluded that SVM was more effective. Yang et al. [14] applied (2D)^2^PCA to finger vein feature extraction and combined it with a KNN classifier based on metric learning for classification, which improved the recognition between different classes of samples. To detect finger vein images more accurately, Qiu et al. [15] proposed a dual-sliding window model and a pseudo-elliptical sampling model to pre-process finger vein images and then used (2D)^2^PCA to extract features from the processed images. Finally, the effectiveness of the method was verified on three datasets. He et al. [16] used PCA for feature dimension reductions and then constructed a multilayer neural network classifier based on the BP neural network. Comparing their results with the literature [13], they noted that the BP neural network classifier took a longer time without significantly improving the recognition accuracy. Ye et al. [17] designed a variable curvature Gabor filter for finger vein orientation feature extraction and then carried out dimensionality reduction by PCA. Hu et al. [18] proposed a block multi-scale uniform local binary pattern (MULBP) method to extract local texture features from the finger vein images, then applied the Two-Dimensional Principal Component Analysis ((2D)^2^PCA) based on block to preserve local features. The average recognition rate of the (2D)^2^PCA tested on different finger vein datasets was over 99%. Ei Wei et al. [19] found that useful local information might be discarded when using PCA for dimensionality reduction. To solve it, they considered that feature extraction using Discrete Wavelet Transform (DWT) with LBP could be used to generate feature vectors before dimensionality reduction using PCA.

According to the results of related literature, it can be found that PCA is a feature extraction method based on the global information of the image. It may regard part of the local key information as noise during the finger vein recognition, which affects the accuracy of finger vein recognition.

LPP is a local feature-based, classical dimensionality reduction algorithm in manifold learning. The method has been used in pattern recognition, face image recognition, and mechanical fault diagnosis but has not yet been applied to finger vein recognition. Due to the similarity in the principle of feature extraction between finger vein recognition and face recognition, LPP is applied to finger vein feature extraction in this paper. He and Niyogi [20] proposed the LPP method in 2004, and later, they applied this method to the study of face recognition in [21]. Gui et al. [22] proposed locality preserving discriminant projections (LPDP) by a adding maximum margin criterion (MMC) to the objective function of LPP and obtained good results in experiments on both the face dataset and palmprint dataset. To alleviate the impact of intra-class variations and improve the performance of gait recognition, Rida et al. [23] proposed a novel method combining the statistical dependency (SD) feature selection with globality-locality preserving projections (GLPP). LPP is sensitive to outliers and noise. Hence, Zhang et al. [24] proposed the sparse locality preserving discriminative projections (SLPDP) method, which combines sparse representation into LPP and verifies the effectiveness of the method on several face datasets. Aminu and Ahmad [25] proposed a locality preserving partial least squares discriminant analysis (LPPLSDA) algorithm that is more suitable for capturing the local structure of faces by adding LPP to partial least squares discriminant analysis (PLSDA) and obtained good performance on several benchmark face databases. However, since features of finger vein pictures are not as rich as fingerprints or palm prints, etc., the success rate of finger vein recognition cannot be effectively improved by simply using LPP.

This paper attempts to combine PCA and LPP more effectively, thus proposing a new finger vein recognition method, namely PCLPP. When using PCLPP for feature extraction of finger vein images, the best projection matrix that preserves both global and local features can be found. The matrix is then used as the input of the SVM classifier, and finally, the recognition rate of finger vein images is improved.

## 3. PCLPP-Based Finger Vein Recognition Framework

**Figure 1:**
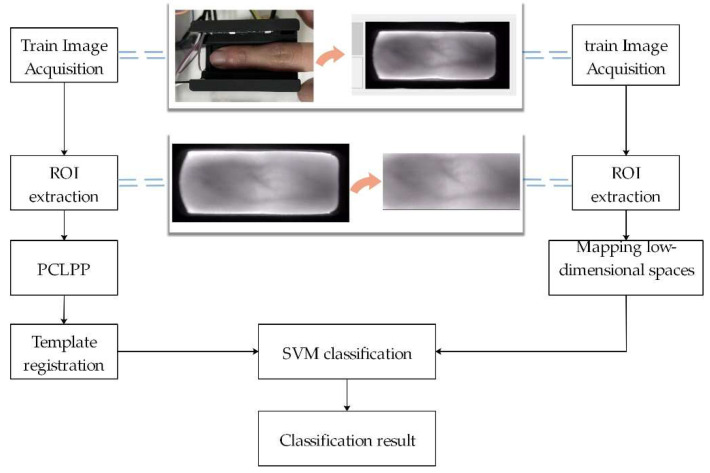
Finger Vein Recognition Framework.

The framework of the PCLPP method proposed in this paper contains two parts, that is, the training part and the testing part, as shown in Figure 1. In the training part, the acquired images are first subjected to a region of interest (ROI) extraction, and then feature extraction is performed by the PCLPP model. The user information is then registered and fed to the SVM classifier for model fitting to obtain an optimal classification hyperplane. In the testing part, the tested images are directly mapped to the low-dimensional space after ROI processing and then fed to the trained SVM model to derive the classification results.

### 3.1. ROI Extraction

**Figure 2:**
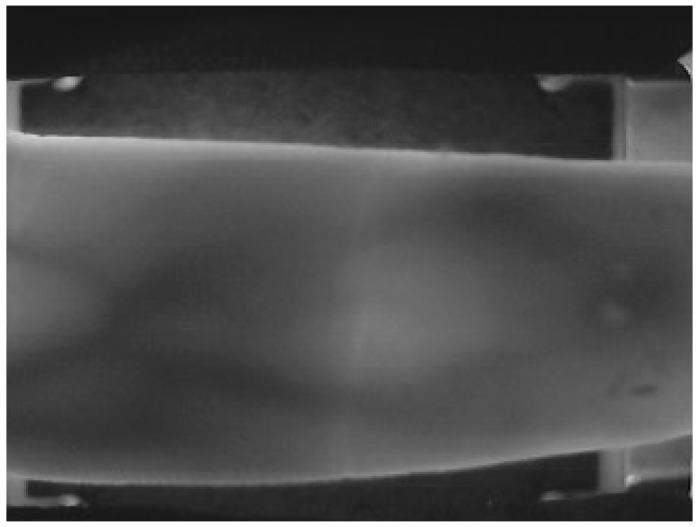
SDUMLA-HMT finger vein imaging map.

Figure 2 shows a finger vein image in SDUMLA-HMT [26]. The irrelevant information such as finger edges and background will interfere with the extraction of finger vein features, which, in turn, affect the finger vein recognition rate [27]. Therefore, to obtain the region containing only finger vein, the ROI extraction for finger vein images is necessary, which is a critical step to enhance the accuracy [28].

### 3.2. PCLPP-Based Feature Extraction Method

Both PCA and LPP can map high-dimensional data to low-dimensional space, but they do so in different ways. PCA aims to preserve the global structure of the image space. It maps high-dimensional data to low-dimensional space by maximizing the global variance. However, it may destroy the internal geometrical structure of the sample dataset because it ignores the correlation between sample points. LPP restores the inherent nonlinear manifold structure of the original map by preserving the local structure, but it does not guarantee the effective preservation of global information, which may lead to the loss of variance information between data points and the destruction of the global structure. To reduce the limitations of both methods, this paper proposed PCLPP, which can retain both the local and the global structure mapping of images, thus achieving the richness and effectiveness of finger vein image feature extraction information.

#### 3.2.1. Notation and Definition

Assume that there is a sample matrix of high-dimensional space $X=\left[x_{1},x_{2},\ldots ,x_{n}\right]\in R^{n\times D}$, where $x_{i}\in R^{D}$, and *D* is the dimensionality of *X*. Consider a linear transformation mapping the original *D*-dimensional space into a *D*-dimensional feature space $Y=\left[y_{1},y_{2},\ldots ,y_{n}\right]\in R^{n\times d}$, where $y_{i}\in R^{d}$ and D≫d. The new feature vectors $y_{i}$ are defined by following linear transformation as Equation (1):

(1) $$y_{i}=P^{T}x_{i},\;i=1,2,\ldots ,n$$

where $P\in R^{n\times d}$ is a transformation matrix.

#### 3.2.2. PCA

PCA is one of the most widely used algorithms for data dimensionality reduction. Its main idea is to map data samples from a high-dimensional space to a low-dimensional space using orthogonal matrices. Its objective function *J*_PCA_ can be formally stated as Equation (2):

(2) $$\begin{array}{c} J_{\mathrm{PCA}}\left(Y\right)=\underset{P}{\max}\overset{n}{\underset{i=1}{\sum }}{∥y_{i}-\bar{y}∥}^{2}\\ s.t.\;P^{T}P=I \end{array}$$

If Equation (1) is used as the conversion equation, Equation (2) can be simplified to the following trace form after a simple algebraic transformation, i.e., Equation (3).

(3) $$\begin{array}{cc} J_{\mathrm{PCA}}\left(P\right) & =\underset{P}{\max}\overset{n}{\underset{i=1}{\sum }}{∥P^{T}\left(x_{i}-\bar{x}\right)∥}^{2}\\ \; & =\underset{P}{\max}\;\mathrm{tr}\{P^{T}\left(x_{i}-\overset{¯}{x}\right){\left(x_{i}-\overset{¯}{x}\right)}^{T}P\}\\ \; & =\underset{P}{\max}\;\mathrm{tr}\left\{P^{T}CP\right\}\\ s.t.\;P^{T}P & =I \end{array}$$

where $C=\sum_{i=1}^{n}\left(x_{i}-\bar{x}\right){\left(x_{i}-\bar{x}\right)}^{T}$ is the covariance matrix, $\bar{x}=\frac{1}{n}\sum_{i=1}^{n}x_{i}$, and tr(*) denotes the trace of matrix *, i.e., the sum of the main diagonal elements of matrix *.

#### 3.2.3. LPP

LPP is a general method for manifold learning and can be considered a linear approximation of the nonlinear Laplacian eigenmaps. LPP keeps the local manifold structure of the data as similar as possible during the transformation of the data projection space. The objective function *J*_LPP_ of LPP can be formally stated as Equation (4):

(4) $$J_{\mathrm{LPP}}\left(P\right)=\underset{P}{\min}\frac{1}{2}\overset{n}{\underset{i,j=1}{\sum }}{∥y_{i}-y_{j}∥}^{2}s_{ij}$$

where *P* is the transformation matrix.

With a simple algebraic reconstruction via Equation (1), the above objective function can be rewritten as follows:

(5) $$\begin{array}{cc} J_{\mathrm{LPP}}\left(P\right) & =\underset{P}{\min}\overset{N}{\underset{i,j=1}{\sum }}\left(P^{T}x_{i}-Px_{j}\right){\left(P^{T}x_{i}-Px_{j}\right)}^{T}s_{ij}\\ \; & =\underset{P}{\min}\;\mathrm{tr}\left\{P^{T}XDX^{T}P-P^{T}XSX^{T}P\right\}\\ \; & =\underset{P}{\min}\;\mathrm{tr}\left\{P^{T}X\left(D-S\right)X^{T}P\right\}\\ \; & =\underset{P}{\min}\;\mathrm{tr}\left\{P^{T}XLX^{T}P\right\}\\ s.t.\;P^{T}XDPX^{T} & =I \end{array}$$

where $D=\mathrm{diag}\left\{D_{11},D_{22},\ldots ,D_{nn}\right\}$ is the diagonal matrix, L=D−S is the Laplacian matrix, and $S={\left[s_{ij}\right]}_{n\times n}$ is the weight matrix obtained with the heat kernel method.

The LPP objective function can therefore be changed to Equation (6).

(6) $$\begin{array}{c} J_{\mathrm{LPP}}\left(P\right)\begin{array}{c} =\underset{P}{\min}\;\mathrm{tr}\left\{P^{T}XLX^{T}P\right\} \end{array}\\ s.t.\;P^{T}XDPX^{T}=I \end{array}$$

#### 3.2.4. PCLPP

Considering that PCA is to find the projection matrix by constructing the global maximized variance, LPP is to obtain the projection matrix by preserving the local features. Therefore, an intuitive motivation is to seek a common projection that minimizes the local scatter *J*_LPP_ while maximizing the global scatter *J*_LPP_. In fact, the optimal projection matrix can be obtained by solving a multi-objective optimization problem, i.e., Equation (7).

(7) $$\begin{array}{c} \left\{\begin{array}{c} \underset{P}{\max}\;\mathrm{tr}\left\{P^{T}CP\right\}\\ \underset{P}{\min}\;\mathrm{tr}\left\{P^{T}XLX^{T}P\right\} \end{array}\right.\\ \begin{array}{c} s.t.\;P^{T}XDX^{T}P=I \end{array} \end{array}$$

PCLPP is seeking the difference between global scattering maximization and local minimization embedding. Therefore, according to the MMC proposed in the literature [29], Equation (7) can be transformed from a multi-objective function to a single objective function, as shown in Equation (8).

(8) $$\begin{array}{c} J_{\mathrm{PCLPP}}=\underset{P}{\max}\;\mathrm{tr}\{P^{T}\left(\alpha C-\left(1-\alpha \right)XLX^{T}\right)P\}\\ s.t.\;P^{T}XDX^{T}P=I \end{array}$$

where $\alpha \in \left[0,1\right)$ is the balance parameter.

To deal with the constraints, we introduced the Lagrange multiplier method to transform Equation (8) into an unconstrained problem, which results in Equation (9).

(9) $$L\left(P\right)=\mathrm{tr}\left\{P^{T}\left(\alpha C-\left(1-\alpha \right)XLX^{T}\right)P\right\}-\lambda_{i}\left(P^{T}XDX^{T}P-I\right)$$

Let $\frac{\partial L\left(P\right)}{\partial P}=0$, we can get

(10) $$\begin{array}{c} \left[\alpha C-\left(1-\alpha \right)XLX^{T}\right]p_{i}=\lambda_{i}XDX^{T}p_{i} \end{array}$$

where $p_{i}$ is the generalized eigenvector corresponding to eigenvalue $\lambda_{i}$.

The steps of the PCLPP algorithm are as follows:

Step 1: Compute *J*_PCA_ and *J*_LPP_, respectively.

Step 2: Construct the overall objective function according to Equation (8) and calculate the eigenvalues and eigenvectors.

Step 3: Select the first *d* feature vectors to form the best feature matrix *P*.

After obtaining the best feature matrix for each finger vein image, the matrices need to be divided into a training set and a test set. Then, the training set is fed into the classifier for training, and the test set is fed into the trained classifier for testing, so that the performance of finger vein image recognition can be evaluated based on the test results. In this paper, the SVM classifier is used for classification and identification.

### 3.3. SVM-Based Image Classification Method

SVM is a commonly used and robust classifier in the field of biometric recognition. Its basic idea is to gain the separation hyperplane that can correctly partition the training data set and maximize the geometric interval. The input of the SVM algorithm should be a set of labeled samples, where $y_{i}\in \left\{+1,-1\right\}$ is the label: +1 for positive cases, and −1 for negative cases.

Then, the mathematical expression of the optimal hyperplane is

(11) $$w^{T}x+b=0$$

where ***x*** is the vector on the hyperplane; $w=\left(w_{1},w_{2},\ldots ,w_{d}\right)$ is the normal vector, which determines the direction of the hyperplane; and *b* is the bias, which determines the distance between the hyperplane and the origin.

The maximum interval is obtained by maximizing the sum of the distance *D* of the two dissimilar support vectors to the hyperplane.

(12) $$\begin{array}{c} D=\underset{w,b}{\max}\frac{2}{\left|\left|w\right|\right|}\\ s.t.\;y_{i}(w^{T}x_{i}+b)\geq 1,\;i=1,2,\ldots ,N \end{array}$$

Combining the Lagrange multiplier method, the convex quadratic programming problem can be transformed into its dual.

(13) $$\begin{array}{l} D^{\prime }=\underset{\alpha }{\max}\overset{m}{\underset{i=1}{\sum }}\alpha_{i}-\frac{1}{2}\overset{N}{\underset{i=1}{\sum }}\overset{N}{\underset{j=1}{\sum }}\alpha_{i}\alpha_{j}y_{i}y_{j}x_{i}^{T}x_{j}\\ s.t.\;\overset{m}{\underset{i=1}{\sum }}\alpha_{i}y_{i}=0,\;\alpha_{i}\geq 0,\;i=1,2,\ldots ,N \end{array}$$

where $\alpha_{i}$, $\alpha_{j}$ are collectively referred to as the dual variables or Lagrange multipliers.

The final decision function of SVM is determined by only a small number of support vectors, so its computational complexity depends on the number of support vectors rather than the dimensionality of the sample space. Therefore, the model can be more robust by adding or deleting non-support vectors. In this paper, the training set images are processed by PCLPP to obtain the optimal training feature matrixes, which are then fed into the SVM classifier to determine the classification criteria. Finally, the low-dimensional matrixes obtained from the test images are fed into the SVM classifier to obtain the classification results according to the classification criteria.

## 4. Experimental Analysis

The SDUMLA-HMT dataset, which is one of the most commonly used datasets in the field of finger vein research, is selected for the experiments in this paper. PCLPP, PCA and LPP, are, respectively, combined with an SVM classifier to perform image recognition experiments on the SDUMLA-HMT dataset, and their performances are compared by recognition accuracy. All the experimental codes were implemented using the Python programming language and were run in a python 3.7 environment. PCA, LPP and PCLPP were implemented by us, while SVM was implemented using the SVM module from the sklearn library. In addition, all experiments were conducted on a desktop computer with an Intel(R) Core(TM) i7-8550U CPU @ 1.80GHz-1.99 GHz and 8 GB RAM.

### 4.1. SDUMLA-HMT Dataset

**Figure 3:**
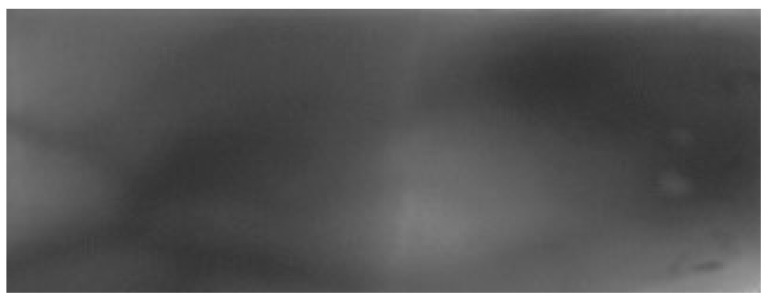
Image after ROI processing.

The SDUMLA-HMT finger vein dataset consisted of 106 subjects, each providing a total of 6 images of the index, middle, and ring fingers of both left and right hands. Thus, 3816 finger images were generated from the SDMULA-HMT dataset, each with 320 × 240 pixels, in ‘bmp’ format. Figure 3 shows the image obtained after ROI extraction, which is 320 × 128 pixels. None of the images were distorted and enhanced during processing.

### 4.2. Finger Vein Recognition Experiments with Different Descending Numbers

**Figure 4:**
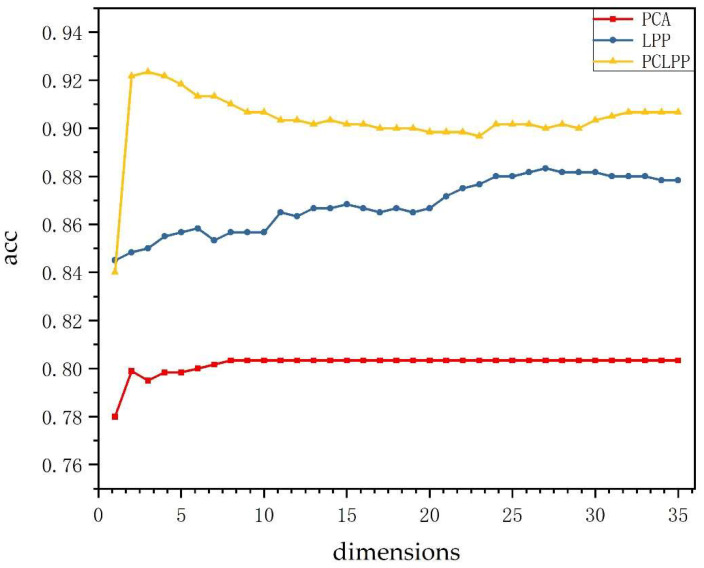
Finger vein recognition rates for PCA, LPP, and PCLPP in different dimensions.

This paper selected 600 classes of finger vein data from the SDUMLA-HMT dataset and divided each class of fingers into training and test sets in the ratio of 5:1. Figure 4 shows the finger vein recognition accuracy in different dimensions by combining the three feature extraction methods (PCA, LPP, and PCLPP) with the SVM classifier, respectively. As can be seen from the figure, the recognition accuracies of the images after feature extraction using PCLPP and LPP both show a trend of increasing and then decreasing with the increase in dimensionality. After using PCLPP for feature extraction, the highest image recognition rate is 0.9233 at dimensions 3. The highest image recognition rate is 0.8833 at dimensions 27 after using LPP. After using PCA for feature extraction, the image recognition rate fluctuates with the increase in dimensionality at first and then stabilizes at 0.8033 after dimensionality reaches 8. Altogether, the recognition accuracy obtained after feature extraction using PCLPP is significantly better than the other two methods under the SDUMLA-HMT dataset. According to the above results, the best dimensionality reduction dimension for each of these three methods can be obtained, and the following experiments are conducted.

### 4.3. Finger Vein Recognition Experiment with Different Category Numbers

**Figure 5:**
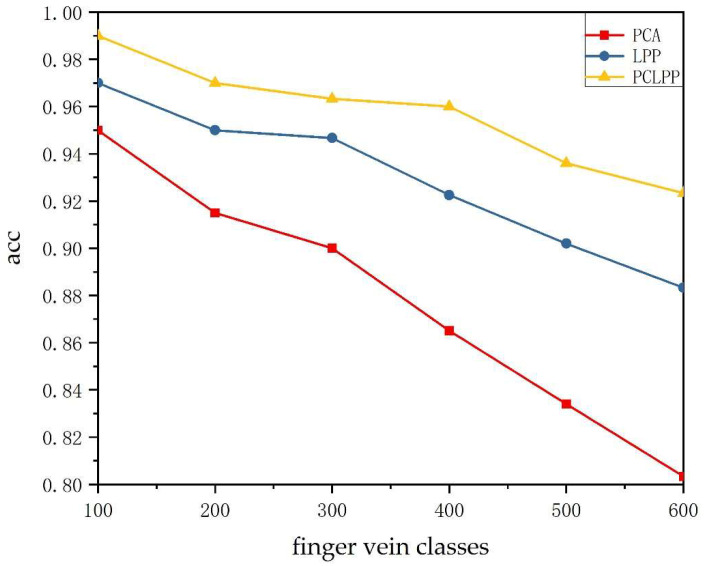
Finger vein recognition rates for PCA, LPP, and PCLPP with different numbers of categories.

Although the topology of finger vein vasculum differs for each category, the similarity of finger vein patterns increases as the number of categories increases. To investigate the relationship between the number of finger vein categories and the three methods, the best dimension reduction for each of these three methods is selected in this paper. The recognition accuracy of 600 categories of fingers in SDUMLA-HMT dataset is tested experimentally once every 100 categories. The experimental results are compiled into Figure 5.

As seen in Figure 5, the finger vein image recognition rate of all three methods after dimensionality reduction to the best dimension tends to decrease as the number of categories increases. PCA, LPP, and PCLPP achieved the best recognition accuracy (0.95, 0.97, 0.98, respectively) for 100 categories of finger vein images and achieved the worst recognition accuracy (0.8033, 0.8817, 0.9233, respectively) for 600 categories of finger vein images. However, PCLPP is significantly better than the other two methods, and its image recognition rate still reaches 0.9233 in 600 categories of fingers. More comprehensively, the recognition rate of the PCLPP method proposed in this paper is significantly better than the other two methods in all categories, and the recognition rate decreases much more slowly than the other two methods as the number of categories increases.

### 4.4. Finger Vein Recognition Experiments after Adding Noise

In the process of finger vein image acquisition, due to the image sensor, transmission channel, decoding processing, etc., bright and dark dot noise will inevitably be generated. These noises will affect the stability and effectiveness of finger vein feature extraction and thus affect the recognition accuracy of finger vein images. In order to verify the robustness of PCLPP to noise, this paper compares the ROI images in the SDUMLA-HMT dataset by adding “Salt and pepper” noise and Gaussian noise.

#### 4.4.1. Adding “Salt and Pepper” Noise

**Figure 6:**
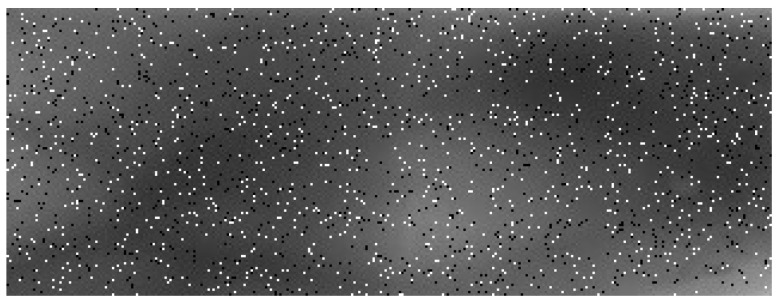
Example of ROI image after setting (0.1) the “Salt and pepper” noise density.

“Salt and pepper” noise is one of the common noises in digital images. Figure 6 shows the ROI image after setting the “Salt and pepper” noise density to 0.1.

**Figure 7:**
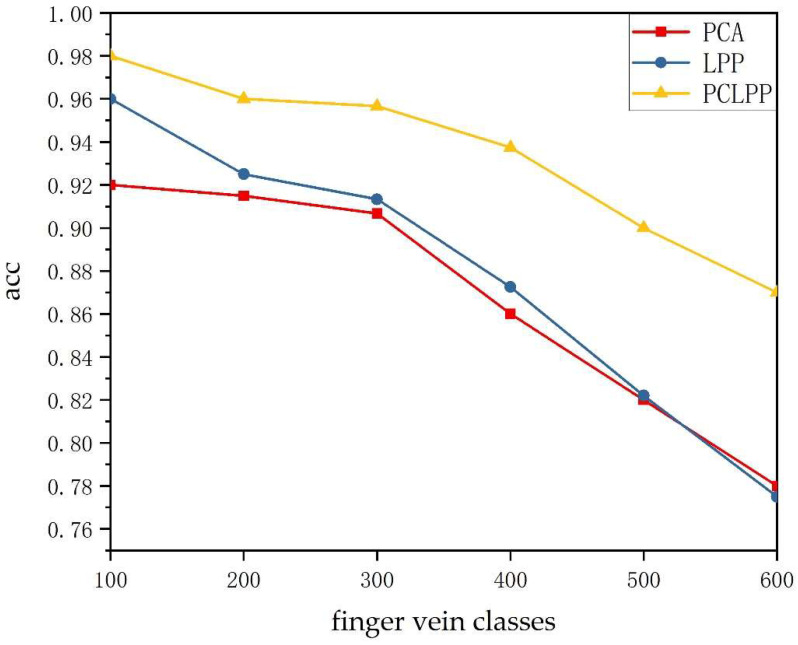
Image recognition rates for PCA, LPP, and PCLPP after adding “Salt and pepper” noise.

Figure 7 shows the image recognition rates of the three methods after adding “Salt and pepper” noise for feature extraction and classification by SVM. As the figure shows, the recognition rate of PCA, LPP, and PCLPP all decrease after adding the “Salt and pepper” noise, but the performance of PCLPP is still significantly better than the other two methods. The recognition rate using PCLPP can still reach 0.870 even in the case of large-capacity (600 classes) finger veins, while the recognition rates of the remaining two methods are lower than 0.800 at this time. The effect of noise on PCA reduction and LPP reduction becomes more and more obvious as the category increases, while the effect on the PCLPP method is relatively small.

#### 4.4.2. Adding Gaussian Noise

**Figure 8:**
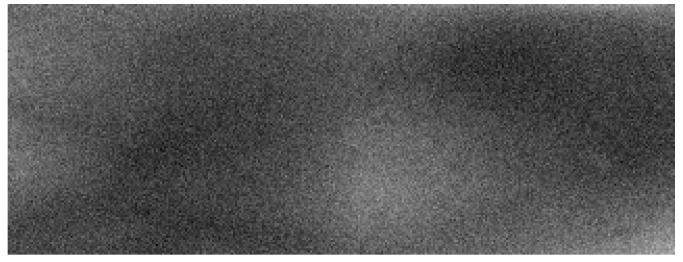
Example of ROI image after setting (0.05) the Gaussian noise variance.

Gaussian noise is also one of the common noises in digital images, and its probability density function obeys the Gaussian distribution. Figure 8 shows the ROI image after setting the Gaussian noise variance to 0.05.

**Figure 9:**
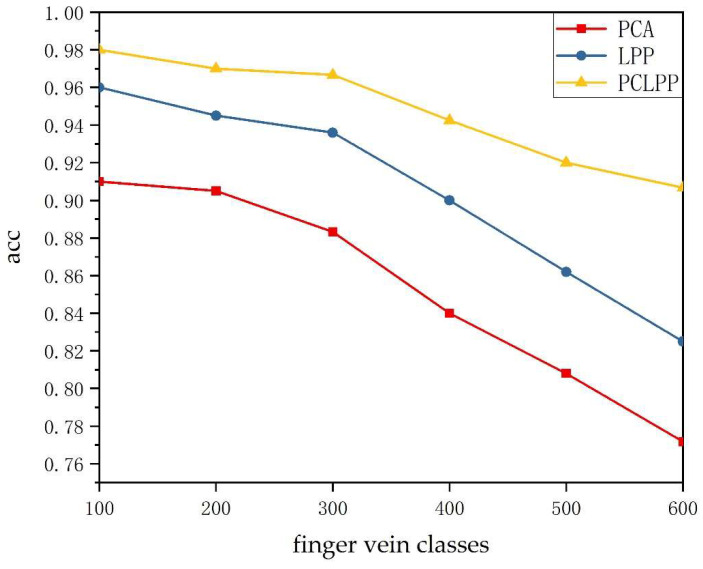
Image recognition rates for PCA, LPP, and PCLPP after adding Gaussian noise.

Figure 9 shows the image recognition rates of the three methods after adding Gaussian noise for feature extraction and classification by SVM. As can be seen, the recognition rates of PCA, LPP, and PCLPP all decrease after adding the “Gaussian noise”, but the performance of PCLPP is still significantly better than the other two methods. The recognition rate using PCLPP can reach 0.907 even in the case of large-capacity (600 classes) finger veins, while the recognition rates of the remaining two methods are lower than 0.830. The effect of noise on PCA reduction and LPP reduction becomes more and more obvious as the category increases, while the effect on the PCLPP method is relatively small.

According to the experimental results in Section 4.4.1 and Section 4.4.2, it can be easily seen that the robustness of PCLPP is better than the other two methods, either by adding “Salt and pepper” noise or Gaussian noise.

We compared the proposed PCLPP with two other methods used in the literature, [1,19], using SDMULA-HMT as the input image set. The identification rates of each method are shown in Table 2. When 600 images were used for the experiments, the recognition accuracy of PCLPP reached 0.99, which was significantly better than that of the literature [19]; when 3600 images were used for the experiments, our method was also better than that of the literature [1].

## 5. Conclusions

In order to solve the problem that traditional finger vein recognition methods cannot obtain discriminative features from finger vein images comprehensively and effectively, this paper proposes a PCLPP algorithm, which organically integrates PCA and LPP and considers both global information and key local information of the image in feature extraction. The image feature extraction by PCLPP can obtain the best projection matrix that preserves both global and local features of the image. Experiments are conducted using the SDUMLA-HMT dataset, and the results show that: (1) the overall finger vein recognition accuracy of classification using the feature matrixes obtained by PCLPP is better than both PCA and LPP methods. (2) After feature extraction by PCLPP, the recognition rate is better than both PCA and LPP methods in a different number of categories. (3) The effect of noise on PCA and LPP is obvious, while the effect on PCLPP is relatively small.

In future research, we will further consider the effects of different image sizes and different classification methods on recognition rates, with the ultimate goal of integrating the finger vein recognition algorithm PCLPP proposed in this paper into a low-cost hardware product (e.g., an access control system with a large number of users).

## Figures and Tables

**Table 1:** Overview of mainstream biometric technologies.

Technology	Accuracy	Stability	Recognition Speed	Convenience	Main Application Drawbacks
Fingerprint	High	Easy to wear and tear	Quick	Low threshold, low cost, easy to use	Small number of users, low accuracy
Face	Average	Average	Slow	Non-contact, easy to collect	Privacy abuse problem, anti-cracking problem
Iris	Extremely high	Very stable	Quick	Non-contact, distance limitation, not easy to collect	High price, high technical difficulty, inconvenient to use
Voice	Average	Unstable	Slow	Easy to acquire, easy to use	Easy to be affected by physical condition and age change, not high in security
Finger vein	Extremely high	Very stable	Quick	Non-contact, easy to acquire	Immature technology, low degree of industrialization

**Table 2:** Identification rate by different methods in SDMULA-HMT.

	Feature Extraction Method	Classification Method	Numbers of Images	Accuracy
[1]	coupled LPQ and LDP	SVM	2860	0.898
[19]	Wavelet + LBP+ PCA	SVM	600	0.9583
Proposed method	PCLPP	SVM	600	0.99
Proposed method	PCLPP	SVM	3600	0.9233

## Data Availability

The data presented in this study are publicly available in the SDUMLA-FV, reference number [26].
